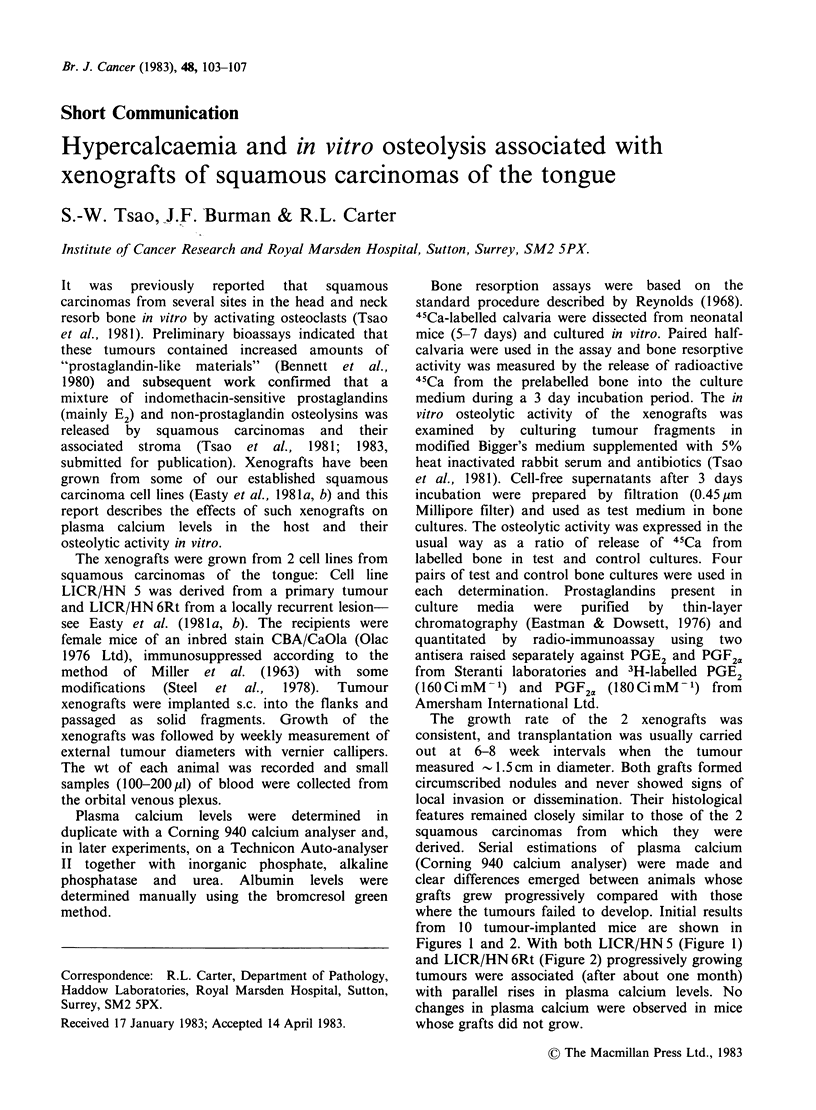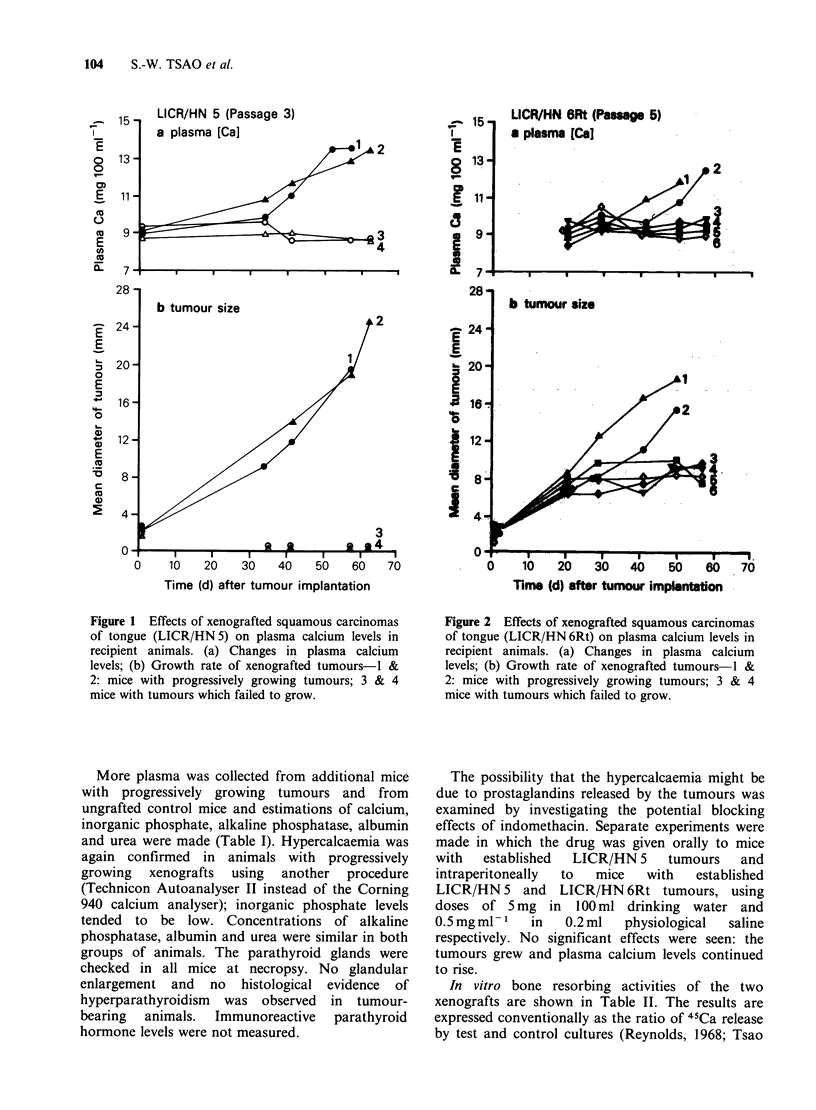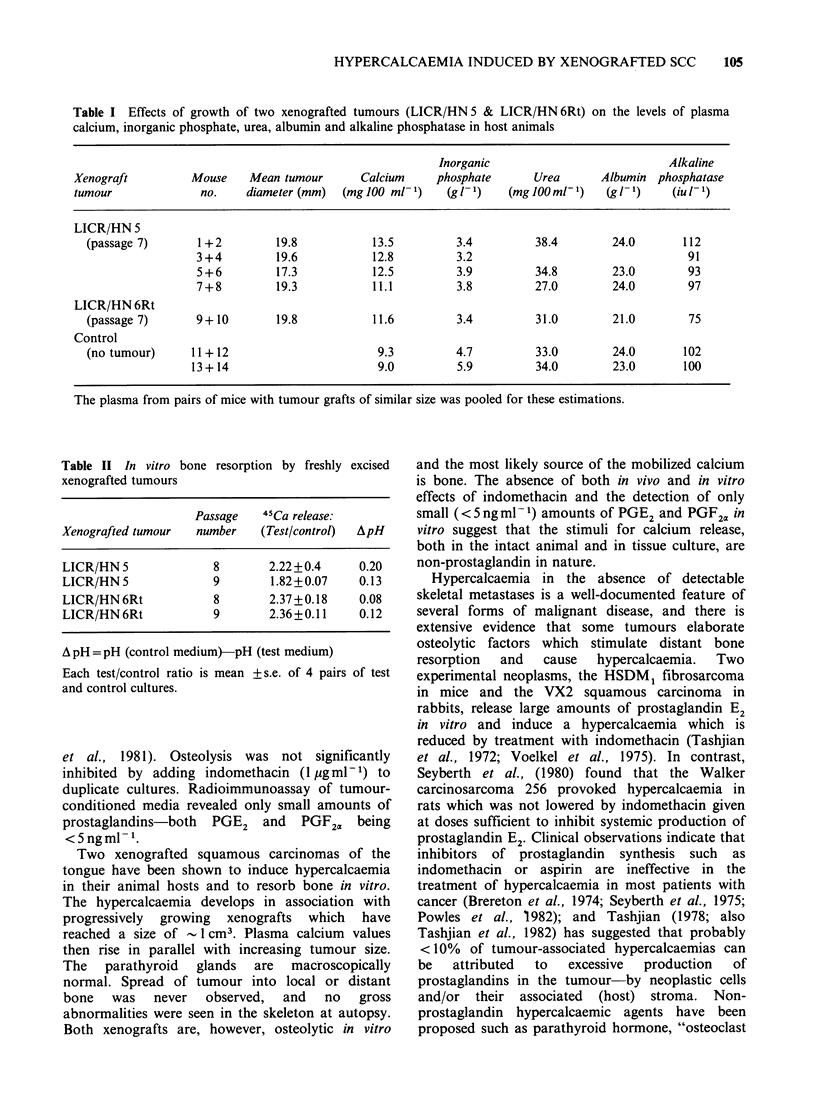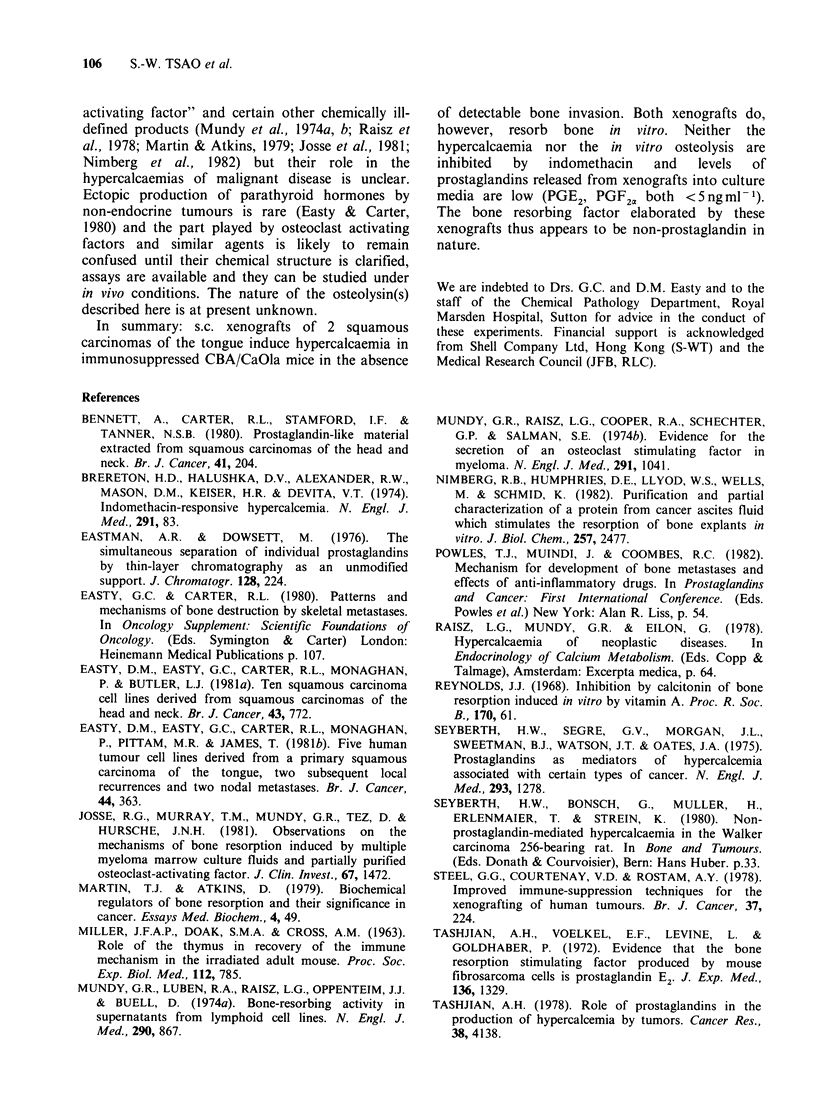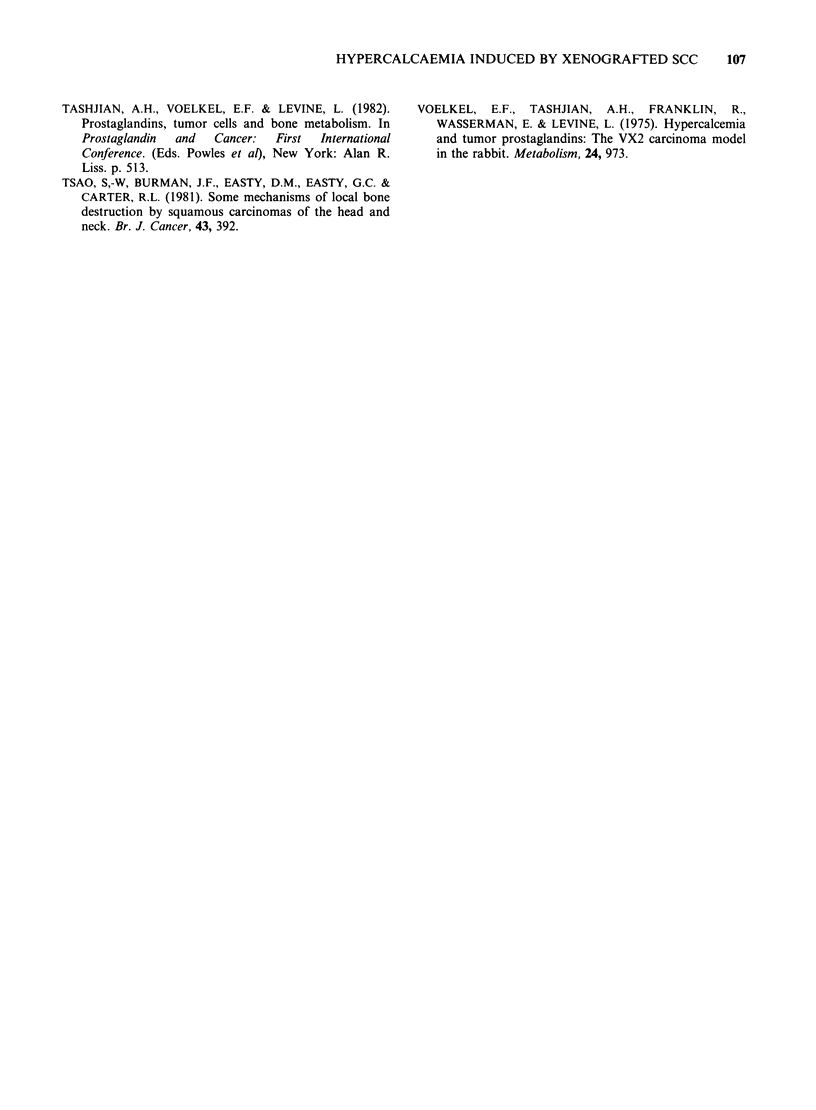# Hypercalcaemia and in vitro osteolysis associated with xenografts of squamous carcinomas of the tongue.

**DOI:** 10.1038/bjc.1983.164

**Published:** 1983-07

**Authors:** S. W. Tsao, J. F. Burman, R. L. Carter


*Br. J. Cancer* (1983), **48**, 103–107

**Short Communication**

### Hypercalcaemia and *in vitro* osteolysis associated with xenografts of squamous carcinomas of the tongue

S.-W. Tsao, J.F. Burman & R.L. Carter

*Institute of Cancer Research and Royal Marsden Hospital, Sutton, Surrey, SM2 5PX.*

It was previously reported that squamous carcinomas from several sites in the head and neck resorb bone *in vitro* by activating osteoclasts (Tsao *et al.*, 1981). Preliminary bioassays indicated that these tumours contained increased amounts of "prostaglandin-like materials" (Bennett *et al.*, 1980) and subsequent work confirmed that a mixture of indomethacin-sensitive prostaglandins (mainly $E_2$) and non-prostaglandin osteolysins was released by squamous carcinomas and their associated stroma (Tsao *et al.*, 1981; 1983, submitted for publication). Xenografts have been grown from some of our established squamous carcinoma cell lines (Easty *et al.*, 1981*a*, *b*) and this report describes the effects of such xenografts on plasma calcium levels in the host and their osteolytic activity *in vitro*.

The xenografts were grown from 2 cell lines from squamous carcinomas of the tongue: Cell line LICR/HN 5 was derived from a primary tumour and LICR/HN 6Rt from a locally recurrent lesion—see Easty *et al.* (1981*a*, *b*). The recipients were female mice of an inbred stain CBA/CaOla (Olac 1976 Ltd), immunosuppressed according to the method of Miller *et al.* (1963) with some modifications (Steel *et al.*, 1978). Tumour xenografts were implanted s.c. into the flanks and passaged as solid fragments. Growth of the xenografts was followed by weekly measurement of external tumour diameters with vernier callipers. The wt of each animal was recorded and small samples (100–200 $\mu$l) of blood were collected from the orbital venous plexus.

Plasma calcium levels were determined in duplicate with a Corning 940 calcium analyser and, in later experiments, on a Technicon Auto-analyser II together with inorganic phosphate, alkaline phosphatase and urea. Albumin levels were determined manually using the bromcresol green method.

Bone resorption assays were based on the standard procedure described by Reynolds (1968). $^{45}$Ca-labelled calvaria were dissected from neonatal mice (5–7 days) and cultured *in vitro*. Paired half-calvaria were used in the assay and bone resorptive activity was measured by the release of radioactive $^{45}$Ca from the prelabelled bone into the culture medium during a 3 day incubation period. The *in vitro* osteolytic activity of the xenografts was examined by culturing tumour fragments in modified Bigger's medium supplemented with 5% heat inactivated rabbit serum and antibiotics (Tsao *et al.*, 1981). Cell-free supernatants after 3 days incubation were prepared by filtration (0.45 $\mu$m Millipore filter) and used as test medium in bone cultures. The osteolytic activity was expressed in the usual way as a ratio of release of $^{45}$Ca from labelled bone in test and control cultures. Four pairs of test and control bone cultures were used in each determination. Prostaglandins present in culture media were purified by thin-layer chromatography (Eastman & Dowsett, 1976) and quantitated by radio-immunoassay using two antisera raised separately against $PGE_2$ and $PGF_{2\alpha}$ from Steranti laboratories and $^3$H-labelled $PGE_2$ (160 Ci mM$^{-1}$) and $PGF_{2\alpha}$ (180 Ci mM$^{-1}$) from Amersham International Ltd.

The growth rate of the 2 xenografts was consistent, and transplantation was usually carried out at 6–8 week intervals when the tumour measured $\sim$1.5 cm in diameter. Both grafts formed circumscribed nodules and never showed signs of local invasion or dissemination. Their histological features remained closely similar to those of the 2 squamous carcinomas from which they were derived. Serial estimations of plasma calcium (Corning 940 calcium analyser) were made and clear differences emerged between animals whose grafts grew progressively compared with those where the tumours failed to develop. Initial results from 10 tumour-implanted mice are shown in Figures 1 and 2. With both LICR/HN 5 (Figure 1) and LICR/HN 6Rt (Figure 2) progressively growing tumours were associated (after about one month) with parallel rises in plasma calcium levels. No changes in plasma calcium were observed in mice whose grafts did not grow.

Correspondence: R.L. Carter, Department of Pathology, Haddow Laboratories, Royal Marsden Hospital, Sutton, Surrey, SM2 5PX.

Received 17 January 1983; Accepted 14 April 1983.

© The Macmillan Press Ltd., 1983

Figure 1 Effects of xenografted squamous carcinomas of tongue (LICR/HN 5) on plasma calcium levels in recipient animals. (a) Changes in plasma calcium levels; (b) Growth rate of xenografted tumours—1 & 2: mice with progressively growing tumours; 3 & 4 mice with tumours which failed to grow.

Figure 2 Effects of xenografted squamous carcinomas of tongue (LICR/HN 6Rt) on plasma calcium levels in recipient animals. (a) Changes in plasma calcium levels; (b) Growth rate of xenografted tumours—1 & 2: mice with progressively growing tumours; 3 & 4 mice with tumours which failed to grow.

More plasma was collected from additional mice with progressively growing tumours and from ungrafted control mice and estimations of calcium, inorganic phosphate, alkaline phosphatase, albumin and urea were made (Table I). Hypercalcaemia was again confirmed in animals with progressively growing xenografts using another procedure (Technicon Autoanalyser II instead of the Corning 940 calcium analyser); inorganic phosphate levels tended to be low. Concentrations of alkaline phosphatase, albumin and urea were similar in both groups of animals. The parathyroid glands were checked in all mice at necropsy. No glandular enlargement and no histological evidence of hyperparathyroidism was observed in tumour-bearing animals. Immunoreactive parathyroid hormone levels were not measured.

The possibility that the hypercalcaemia might be due to prostaglandins released by the tumours was examined by investigating the potential blocking effects of indomethacin. Separate experiments were made in which the drug was given orally to mice with established LICR/HN 5 tumours and intraperitoneally to mice with established LICR/HN 5 and LICR/HN 6Rt tumours, using doses of 5 mg in 100 ml drinking water and 0.5 mg ml$^{-1}$ in 0.2 ml physiological saline respectively. No significant effects were seen: the tumours grew and plasma calcium levels continued to rise.

*In vitro* bone resorbing activities of the two xenografts are shown in Table II. The results are expressed conventionally as the ratio of $^{45}Ca$ release by test and control cultures (Reynolds, 1968; Tsao

**Table I** Effects of growth of two xenografted tumours (LICR/HN 5 & LICR/HN 6Rt) on the levels of plasma calcium, inorganic phosphate, urea, albumin and alkaline phosphatase in host animals

| *Xenograft tumour* | *Mouse no.* | *Mean tumour diameter (mm)* | *Calcium* ($mg\ 100\ ml^{-1}$) | *Inorganic phosphate* ($g\ l^{-1}$) | *Urea* ($mg\ 100\ ml^{-1}$) | *Albumin* ($g\ l^{-1}$) | *Alkaline phosphatase* ($iu\ l^{-1}$) |
|---|---|---|---|---|---|---|---|
| LICR/HN 5 | | | | | | | |
| (passage 7) | 1+2 | 19.8 | 13.5 | 3.4 | 38.4 | 24.0 | 112 |
| | 3+4 | 19.6 | 12.8 | 3.2 | | | 91 |
| | 5+6 | 17.3 | 12.5 | 3.9 | 34.8 | 23.0 | 93 |
| | 7+8 | 19.3 | 11.1 | 3.8 | 27.0 | 24.0 | 97 |
| LICR/HN 6Rt | | | | | | | |
| (passage 7) | 9+10 | 19.8 | 11.6 | 3.4 | 31.0 | 21.0 | 75 |
| Control | | | | | | | |
| (no tumour) | 11+12 | | 9.3 | 4.7 | 33.0 | 24.0 | 102 |
| | 13+14 | | 9.0 | 5.9 | 34.0 | 23.0 | 100 |

The plasma from pairs of mice with tumour grafts of similar size was pooled for these estimations.

**Table II** *In vitro* bone resorption by freshly excised xenografted tumours

| *Xenografted tumour* | *Passage number* | $^{45}$*Ca release: (Test/control)* | $\Delta pH$ |
|---|---|---|---|
| LICR/HN 5 | 8 | 2.22±0.4 | 0.20 |
| LICR/HN 5 | 9 | 1.82±0.07 | 0.13 |
| LICR/HN 6Rt | 8 | 2.37±0.18 | 0.08 |
| LICR/HN 6Rt | 9 | 2.36±0.11 | 0.12 |

$\Delta$pH = pH (control medium)—pH (test medium)

Each test/control ratio is mean ±s.e. of 4 pairs of test and control cultures.

*et al.*, 1981). Osteolysis was not significantly inhibited by adding indomethacin (1 $\mu g\ ml^{-1}$) to duplicate cultures. Radioimmunoassay of tumour-conditioned media revealed only small amounts of prostaglandins—both $PGE_2$ and $PGF_{2\alpha}$ being $<5\ ng\ ml^{-1}$.

Two xenografted squamous carcinomas of the tongue have been shown to induce hypercalcaemia in their animal hosts and to resorb bone *in vitro*. The hypercalcaemia develops in association with progressively growing xenografts which have reached a size of $\sim 1\ cm^3$. Plasma calcium values then rise in parallel with increasing tumour size. The parathyroid glands are macroscopically normal. Spread of tumour into local or distant bone was never observed, and no gross abnormalities were seen in the skeleton at autopsy. Both xenografts are, however, osteolytic *in vitro* and the most likely source of the mobilized calcium is bone. The absence of both *in vivo* and *in vitro* effects of indomethacin and the detection of only small ($<5\ ng\ ml^{-1}$) amounts of $PGE_2$ and $PGF_{2\alpha}$ *in vitro* suggest that the stimuli for calcium release, both in the intact animal and in tissue culture, are non-prostaglandin in nature.

Hypercalcaemia in the absence of detectable skeletal metastases is a well-documented feature of several forms of malignant disease, and there is extensive evidence that some tumours elaborate osteolytic factors which stimulate distant bone resorption and cause hypercalcaemia. Two experimental neoplasms, the $HSDM_1$ fibrosarcoma in mice and the VX2 squamous carcinoma in rabbits, release large amounts of prostaglandin $E_2$ *in vitro* and induce a hypercalcaemia which is reduced by treatment with indomethacin (Tashjian *et al.*, 1972; Voelkel *et al.*, 1975). In contrast, Seyberth *et al.*, (1980) found that the Walker carcinosarcoma 256 provoked hypercalcaemia in rats which was not lowered by indomethacin given at doses sufficient to inhibit systemic production of prostaglandin $E_2$. Clinical observations indicate that inhibitors of prostaglandin synthesis such as indomethacin or aspirin are ineffective in the treatment of hypercalcaemia in most patients with cancer (Brereton *et al.*, 1974; Seyberth *et al.*, 1975; Powles *et al.*, 1982); and Tashjian (1978; also Tashjian *et al.*, 1982) has suggested that probably <10% of tumour-associated hypercalcaemias can be attributed to excessive production of prostaglandins in the tumour—by neoplastic cells and/or their associated (host) stroma. Non-prostaglandin hypercalcaemic agents have been proposed such as parathyroid hormone, "osteoclast activating factor" and certain other chemically ill-defined products (Mundy *et al.*, 1974*a*, *b*; Raisz *et al.*, 1978; Martin & Atkins, 1979; Josse *et al.*, 1981; Nimberg *et al.*, 1982) but their role in the hypercalcaemias of malignant disease is unclear. Ectopic production of parathyroid hormones by non-endocrine tumours is rare (Easty & Carter, 1980) and the part played by osteoclast activating factors and similar agents is likely to remain confused until their chemical structure is clarified, assays are available and they can be studied under *in vivo* conditions. The nature of the osteolysin(s) described here is at present unknown.

In summary: s.c. xenografts of 2 squamous carcinomas of the tongue induce hypercalcaemia in immunosuppressed CBA/CaOla mice in the absence of detectable bone invasion. Both xenografts do, however, resorb bone *in vitro*. Neither the hypercalcaemia nor the *in vitro* osteolysis are inhibited by indomethacin and levels of prostaglandins released from xenografts into culture media are low ($PGE_2$, $PGF_{2\alpha}$ both $<5\,ng\,ml^{-1}$). The bone resorbing factor elaborated by these xenografts thus appears to be non-prostaglandin in nature.

We are indebted to Drs. G.C. and D.M. Easty and to the staff of the Chemical Pathology Department, Royal Marsden Hospital, Sutton for advice in the conduct of these experiments. Financial support is acknowledged from Shell Company Ltd, Hong Kong (S-WT) and the Medical Research Council (JFB, RLC).

### References

BENNETT, A., CARTER, R.L., STAMFORD, I.F. & TANNER, N.S.B. (1980). Prostaglandin-like material extracted from squamous carcinomas of the head and neck. *Br. J. Cancer*, **41**, 204.

BRERETON, H.D., HALUSHKA, D.V., ALEXANDER, R.W., MASON, D.M., KEISER, H.R. & DEVITA, V.T. (1974). Indomethacin-responsive hypercalcemia. *N. Engl. J. Med.*, **291**, 83.

EASTMAN, A.R. & DOWSETT, M. (1976). The simultaneous separation of individual prostaglandins by thin-layer chromatography as an unmodified support. *J. Chromatogr.* **128**, 224.

EASTY, G.C. & CARTER, R.L. (1980). Patterns and mechanisms of bone destruction by skeletal metastases. In *Oncology Supplement: Scientific Foundations of Oncology*. (Eds. Symington & Carter) London: Heinemann Medical Publications p. 107.

EASTY, D.M., EASTY, G.C., CARTER, R.L., MONAGHAN, P. & BUTLER, L.J. (1981*a*). Ten squamous carcinoma cell lines derived from squamous carcinomas of the head and neck. *Br. J. Cancer*, **43**, 772.

EASTY, D.M., EASTY, G.C., CARTER, R.L., MONAGHAN, P., PITTAM, M.R. & JAMES, T. (1981*b*). Five human tumour cell lines derived from a primary squamous carcinoma of the tongue, two subsequent local recurrences and two nodal metastases. *Br. J. Cancer*, **44**, 363.

JOSSE, R.G., MURRAY, T.M., MUNDY, G.R., TEZ, D. & HURSCHE, J.N.H. (1981). Observations on the mechanisms of bone resorption induced by multiple myeloma marrow culture fluids and partially purified osteoclast-activating factor. *J. Clin. Invest.*, **67**, 1472.

MARTIN, T.J. & ATKINS, D. (1979). Biochemical regulators of bone resorption and their significance in cancer. *Essays Med. Biochem.*, **4**, 49.

MILLER, J.F.A.P., DOAK, S.M.A. & CROSS, A.M. (1963). Role of the thymus in recovery of the immune mechanism in the irradiated adult mouse. *Proc. Soc. Exp. Biol. Med.*, **112**, 785.

MUNDY, G.R., LUBEN, R.A., RAISZ, L.G., OPPENTEIM, J.J. & BUELL, D. (1974*a*). Bone-resorbing activity in supernatants from lymphoid cell lines. *N. Engl. J. Med.*, **290**, 867.

MUNDY, G.R., RAISZ, L.G., COOPER, R.A., SCHECHTER, G.P. & SALMAN, S.E. (1974*b*). Evidence for the secretion of an osteoclast stimulating factor in myeloma. *N. Engl. J. Med.*, **291**, 1041.

NIMBERG, R.B., HUMPHRIES, D.E., LLYOD, W.S., WELLS, M. & SCHMID, K. (1982). Purification and partial characterization of a protein from cancer ascites fluid which stimulates the resorption of bone explants *in vitro*. *J. Biol. Chem.*, **257**, 2477.

POWLES, T.J., MUINDI, J. & COOMBES, R.C. (1982). Mechanism for development of bone metastases and effects of anti-inflammatory drugs. In *Prostaglandins and Cancer: First International Conference*. (Eds. Powles *et al.*) New York: Alan R. Liss, p. 54.

RAISZ, L.G., MUNDY, G.R. & EILON, G. (1978). Hypercalcaemia of neoplastic diseases. In *Endocrinology of Calcium Metabolism*. (Eds. Copp & Talmage), Amsterdam: Excerpta medica, p. 64.

REYNOLDS, J.J. (1968). Inhibition by calcitonin of bone resorption induced *in vitro* by vitamin A. *Proc. R. Soc. B.*, **170**, 61.

SEYBERTH, H.W., SEGRE, G.V., MORGAN, J.L., SWEETMAN, B.J., WATSON, J.T. & OATES, J.A. (1975). Prostaglandins as mediators of hypercalcemia associated with certain types of cancer. *N. Engl. J. Med.*, **293**, 1278.

SEYBERTH, H.W., BONSCH, G., MULLER, H., ERLENMAIER, T. & STREIN, K. (1980). Non-prostaglandin-mediated hypercalcaemia in the Walker carcinoma 256-bearing rat. In *Bone and Tumours*. (Eds. Donath & Courvoisier), Bern: Hans Huber. p.33.

STEEL, G.G., COURTENAY, V.D. & ROSTAM, A.Y. (1978). Improved immune-suppression techniques for the xenografting of human tumours. *Br. J. Cancer*, **37**, 224.

TASHJIAN, A.H., VOELKEL, E.F., LEVINE, L. & GOLDHABER, P. (1972). Evidence that the bone resorption stimulating factor produced by mouse fibrosarcoma cells is prostaglandin $E_2$. *J. Exp. Med.*, **136**, 1329.

TASHJIAN, A.H. (1978). Role of prostaglandins in the production of hypercalcemia by tumors. *Cancer Res.*, **38**, 4138.

TASHJIAN, A.H., VOELKEL, E.F. & LEVINE, L. (1982). Prostaglandins, tumor cells and bone metabolism. In *Prostaglandin and Cancer: First International Conference*. (Eds. Powles *et al*), New York: Alan R. Liss. p. 513.

TSAO, S,-W, BURMAN, J.F., EASTY, D.M., EASTY, G.C. & CARTER, R.L. (1981). Some mechanisms of local bone destruction by squamous carcinomas of the head and neck. *Br. J. Cancer,* **43,** 392.

VOELKEL, E.F., TASHJIAN, A.H., FRANKLIN, R., WASSERMAN, E. & LEVINE, L. (1975). Hypercalcemia and tumor prostaglandins: The VX2 carcinoma model in the rabbit. *Metabolism,* **24,** 973.